# Protecting the Zaporizhzhia nuclear plant: A call for global action!

**DOI:** 10.1002/puh2.23

**Published:** 2022-11-04

**Authors:** Ataman Yurii, Deborah Oluwaseun Shomuyiwa, Albina Zharkova, Kolesnyk Pavlo

**Affiliations:** ^1^ Rehabilitation Department Sumy State University Sumy Ukraine; ^2^ Faculty of Pharmacy University of Lagos Lagos Nigeria; ^3^ Department of Family Medicine Sumy State University Sumy Ukraine; ^4^ Department of Family Medicine and Outpatient Care Uzhhorod State University Uzhhorod Ukraine

The Russian invasion of Ukraine has seen an intense power trip, with the Zaporizhzhia nuclear power plant at the centre of it. The six gigawatt (GW) plant accounts for 20% of Ukraine's annual electricity supply and can power up to four million homes [1]. Located about 200 km from the heavily contested Donbas region and the Russia‐annexed Crimea, the plant generates 40% of Ukraine's nuclear‐derived power. The Ukrainian Armed Forces reported that the Russian military occupying the nuclear power plant since March 2022, operates the plant as a military base and as a shield [2]. While it has been implied that the Russian forces want to connect the plant to the Crimean electricity grid, there is no indication that they will not damage or destroy the plant. The largest nuclear power plant in Europe and the fifth largest in the world has been subject to repeated shelling by Russian forces and reported rocket strikes leading to explosions and ‘accidental fire outbreaks’ [3]. This has called for warnings of an impending ‘nuclear disaster’ [2].

The nuclear reactors, fuelled by Uranium‐235 to produce heat for electricity generation, are at the centre of the nuclear plant and are continually cooled by offsite power generators [1]. With damage to the plant's nitrogen‐oxygen station and attacks on the processed fuel stored outside the plant, military action is jeopardising the overall security of the power plant [2]. Proper cooling of the facility is integral, as reactors losing cooling facilities creates high pressure and increases the risk of explosion and radioactive leaks. Radioactive poisoning and cancers, among other illnesses, are characteristic effects of radioactive leaks. Damaged nitrogen‐oxygen units with constant infrastructure damage increase the risk of hydrogen leakage and emission of radioactive substances. Radiation leakages have unpredictable and long‐term effects with no geographic or time limitations. Europe is at risk due to the Zaporizhzhia nuclear plant's prime location in the middle of the continent. A nuclear accident at the plant will result in a disaster bigger than that of Chernobyl in 1986 causing a global security emergency [4].

This compounding fear of nuclear disaster calls for a need for pre‐emptive precautionary measures. The Russian war on Ukraine has seen several human rights infringements, food insecurity, increasing numbers of displaced persons and a growing energy crisis. All these developments are occurring in the backdrop of extreme weather and heat waves resulting in several cases of wildfires in its record hottest summer in Europe in 2022. Nuclear‐powered electricity is one of the clean energy sources and helps fight climate change. The attacks on the plant represent attacks on the global nuclear energy space and efforts to combat climate change [5].

The risk of further attacks on the plant is too great and unacceptable for population safety, especially with the broken talks on ending the war. With parts of the plant getting in the line of fire, the United Nations, through the International Atomic Energy Agency (IAEA), has urged utmost restraint around the site as any mishap could lead to potential nuclear damage [4]. Several factors are integrated with nuclear instability, including the physical integrity of the plant, logistics of nuclear material, and supply chain interruption. The IAEA needs to be granted access immediately for expert technical inspection and validation of the nuclear plant's safety. This is important for emergency preparedness as well as to address safety concerns. With the indispensable pillars of nuclear safety and security compromised, contrary to Moscow's assertion [6], the IAEA must be allowed to conduct the exercise immediately.

A pushback against Russian activities is vital for the control of military activities. The absolute withdrawal of Russian troops from the nuclear facility is integral to ensuring the safety of the infrastructure and, by extension, the safety of Europe. Demilitarisation of the zone of the nuclear plant and its surrounding areas involves the control of the area back to Ukraine. The use of the nuclear power plant as a hostage and measure of threat is synonymous with nuclear terrorism. The withdrawal of the Russian military should set the pace for the end of the war. The Russian forces must understand that the unfounded war and dire conditions at the Zaporizhzhia nuclear plant, mediated by the war, pose a significant risk to international peace and security. Military activities must end at the nuclear site, and its surroundings and room must be given to re‐enact safe operations at the Zaporizhzhia nuclear plant.

The European Union and United Nations, major European and global peace and security stakeholders, must move beyond sanctions and strong condemnations to prompt action. Technical support, as well as the continued physical presence of the IAEA, is critical for emergency preparedness and response. Protecting the power supply of the plant, as well as providing alternative sources, is critical to the plant's safe operation. Increased surveillance is essential to track the security and stability of nuclear reactors. While the Ukrainian resistance is growing and the Russian encampments are becoming targets for Ukrainian forces, strikes in the facility represent a huge risk to the facility. Global and strategic cooperation, especially amongst nuclear states, can drive Russia's withdrawal from the nuclear plant. With the Chernobyl nuclear disaster as a reference, the United Nations Security Council must lead in action and mobilise a global response.

## AUTHOR CONTRIBUTIONS

Ataman Yurii: Writing – review & editing. Deborah Oluwaseun Shomuyiwa: Conceptualization; Writing – original draft; Writing – review & editing. Albina Zharkova: Writing – review & editing. Kolesnyk Pavlo: Writing – review & editing

## FUNDING STATEMENT

There is no funding for the development of this paper.

## CONFLICT OF INTEREST

Deborah Oluwaseun Shomuyiwa is a Youth Editorial Board member of Public Health Challenges and a co‐author of this article. To minimize bias, she has been excluded from all editorial decision‐making related to the acceptance of this article for publication.

## ETHICS APPROVAL

Ethics approval is not required for this type of article.

## Data Availability

No database or primary data was used in preparing the manuscript.
